# Functional verification and screening of protein interacting with the slPHB3

**DOI:** 10.1080/15592324.2022.2025678

**Published:** 2022-02-03

**Authors:** Haining Li, Yitong Mu, Xu Chang, GuanRong Li, Zhongquan Dong, Jun Sun, Shengxuan Jin, Xiaolu Wang, Ling Zhang, Shumei Jin

**Affiliations:** a Key Laboratory of Saline-alkali Vegetation Ecology Restoration, Ministry of Education, College of Life Sciences, Northeast Forestry University, Harbin, China; b College of Forestry, Northeast Forestry University, Harbin, China

**Keywords:** Salix linearistipularis, PHB3, salt, yeast two-hybrid

## Abstract

*slPHB3* was cloned from *Salix linearistipularis*, the amino acid sequence blast and phylogenetic tree analysis showed that slPHB3 has the most similarity with PHB3 from *Populus trichocarpa* using DNAMAN software and MEGA7 software. RT-qPCR results confirmed that the expression of *slPHB3* was induced obviously under stress treatments. The growth of recombinant yeast cells was better than that of the control group under the stress treatment, indicating that *slPHB3* may be involved in the stress response of yeast cells. The transgenic tobacco was treated with different concentrations of NaCl, NaHCO_3_ and H_2_O_2_, fresh weigh of overexpression tobacco were heavier than wild-types. The results showed that transgenic tobacco was more tolerant to salt and oxidation than wild-type tobacco. Expression of important genes including *NHX1* and *SOS1* in salt stress response pathways are steadily higher in overexpression tobacco than that in wild-types. We identified 17 proteins interacting with slPHB3 by yeast two-hybrid technique, most of these proteins were relation to the stresses. The salt tolerance of *slPHB3* expressing yeast and *slPHB3* overexpressing plants were better than that of the control. Ten stress-related proteins may interact with slPHB3, which preliminarily indicated that *slPHB3* had a certain response relationship with salt stress. The study of *slPHB3* under abiotic stress can improve our understanding of *PHB3* gene function.

## Introduction

Plants have developed specific mechanisms that allow them to detect precise environmental changes and respond to complex stress conditions.^1^ *Salix linearistipularis* (syn. S. mongolica) is a woody plant that is found in Songnen plain, Heilongjiang, China.^2^ *Salix linearistipularis* resistant to saline and plays an important role in maintaining ecological balance and improving saline soil.^3^

*PHB* was first discovered as a tumor suppressor gene in mammalian cells.^4^ *PHB* genes are conserved during evolution.^5,6^ PHB protein regulates plant growth, regulate membrane protein degradation, control cell proliferation and apoptosis.^7,8^ *PHB3* impacts SA accumulation.^9^ *PHB3* knockout mutants show severe growth defects and have decreased cell division and expansion in the root apex.^10^ *PHB3* regulates stem cell niche maintenance and cell proliferation during root development in *Arabidopsis*.^11^ Germination of *atPHB3* KO seeds was delayed by 1–2 days compared with wild-type.^12^ Methylo bacterium has better survival ability under osmotic stress, which is related to the accumulation of *PHB* in the strain.^13^In *Arabidopsis thaliana*, PHB3 coordinates cell division and differentiation in root tip meristems by restricting the ethylene reactive factor transcription factor.^14^ The growth of wildtype was different from that of the *PHB3* mutant under NaCl or H_2_O_2_ treatment.^10^

Salinity stress effect on plant can be reduced by maintaining the appropriate Na^+^ ions concentration in the cytosol, *NHX1* (vacuolar Na^+^/H^+^ antiporter) and *SOS1* (Salt Overly-Sensitive-1, plasma membrane Na^+^/H^+^ antiporter 1) are a set of genes that function in ion homeostasis in plants by regulating Na^+^ ions in the cytosol. NHX1 are present in tonoplasts and decrease the accumulation of Na^+^ ions in the cytosol by pumping Na^+^ in the vacuole to sequester Na^+^ in vacuoles, SOS1 is located at the plasma membrane and is responsible for efflux Na^+^ from cytosol to apoplasts,^15^ *NHX1*and *SOS1* might be used as biomarkers to further investigate the interaction between gene and stress tolerance.^16^ *SpSOS1* transgenic plants might have a high capacity for removing ROS under salt stress.^17^ *TdSOS1* transgenic plants have higher ROS scavenging enzymes activity than that in control plants under oxidative stress.^18^ Co-expression of Na^+^/H^+^ antiporter *NHX1* and DEAD-box RNA helicase *eIF4A1* from Arabidopsis positively regulates drought stress tolerance by improving ROS scavenging capacity.^19^

In this study, *slPHB3* was cloned from *Salix linearistipularis*, then transferred into yeast and tobacco genome. The results showed that transgenic tobacco plants had stronger salt tolerance and oxidation resistance than wild-type tobacco. Seventeen positive clones were obtained using yeast two-hybrid assay to further explore the interaction protein with slPHB3. *slPHB3* enhanced the stress resistance of transgenic tobacco plants and provided a basis for the study of *PHB3* introduction in other valuable plants.

## Result

### *Cloning and bioinformatics analysis of* slPHB3 *gene*

**Figure 1.**
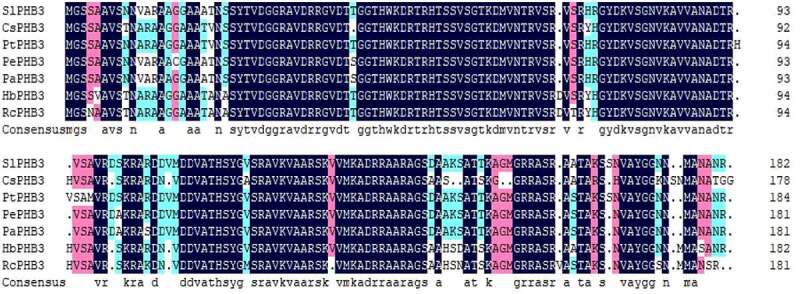
Alignment of the slPHB3 amino acid sequence with PHB3 proteins from other plant species. The amino acid sequence is similar to that of CsPHB3 Protein (XP_006469979.1), PtPHB3 Protein (XP_002323792.1), PePHB3 Protein (XP_011045196.1), PaPHB3 protein (TKR74705.1), HbPHB3 Protein (XP_021677719.1), RcPHB3 protein(XP_002509571.1).

The open reading frame sequence of the *slPHB3* gene was cloned from *Salix linearistipularis* and sequenced. PHB3 protein of other plants was compared with slPHB3 by DNAMan software. The amino acid sequence of slPHB3 has a high degree similarity with the amino acid sequence of other plants PHB3 (Figure 1). Therefore, the cDNA cloned from *Salix linearistipularis* is named as *slPHB3*.

**Figure 2.**
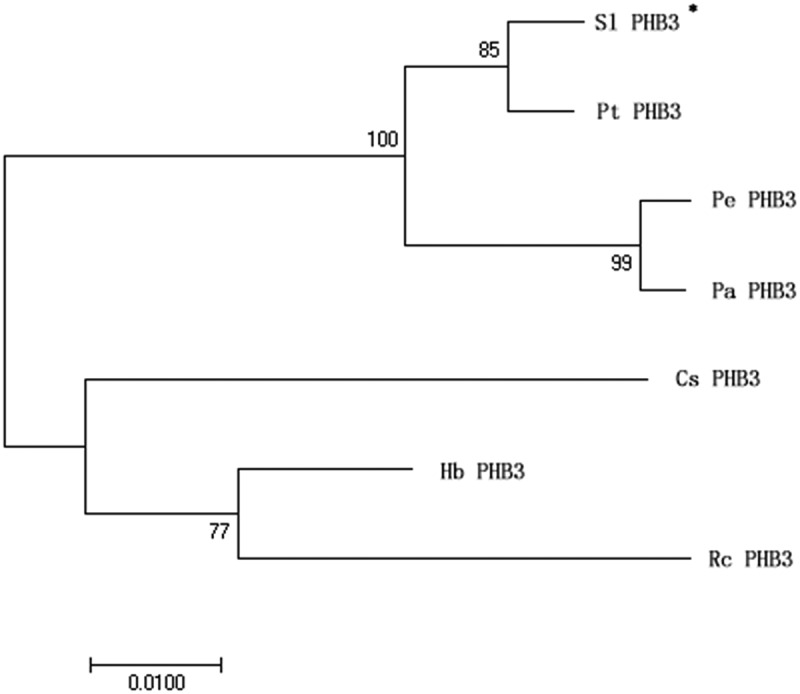
*slPHB3* evolutionary tree analysis. The MEGA7 program was used for the construction of phylogenetic trees. Bar represents 0.1 amino acid substitutions per site.

In order to explore the amino acid sequence homology between slPHB3 and other plant PHB3, we constructed a phylogenetic tree (Figure 2), which showed that slPHB3 is closely related to PHB3 from Populus trichocarpa, Populus euphratica, and Populus alba.

### *Expression of* slPHB3 *in* salix linearistipularis *under abiotic stresses*

**Figure 3.**
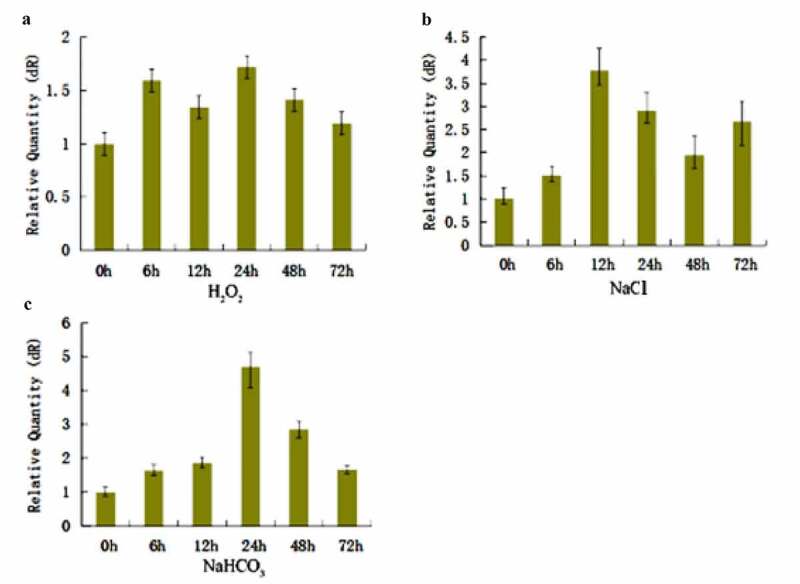
Real-time quantitative PCR analysis for *slPHB3* expression in *Salix linearistipularis* under different stresses. (a): Relative expression of *slPHB3* at different time under 3 mM H_2_O_2_ stress; (b): Relative expression of *slPHB3* at different time under 125 mM NaCl stress; (c): Relative expression of *slPHB3* at different time under 5 mM NaHCO_3_ stress.

The *slPHB3* expression level in the *Salix linearistipularis* leaves increased until the highest level at 24 h and then decreased gradually under 3 mM H_2_O_2_ and 5mM NaHCO_3_ stress (Figure 3a). The *slPHB3* expression level showed the highest value at 12 h under 125 mM NaCl stress (Figure 3b and c). That means that the stresses influenced the *slPHB3* gene expression level in *Salix linearistipularis* leaves.

### *Expression of* slPHB3 *gene in transgenic yeast in response to stresses*

**Figure 4.**
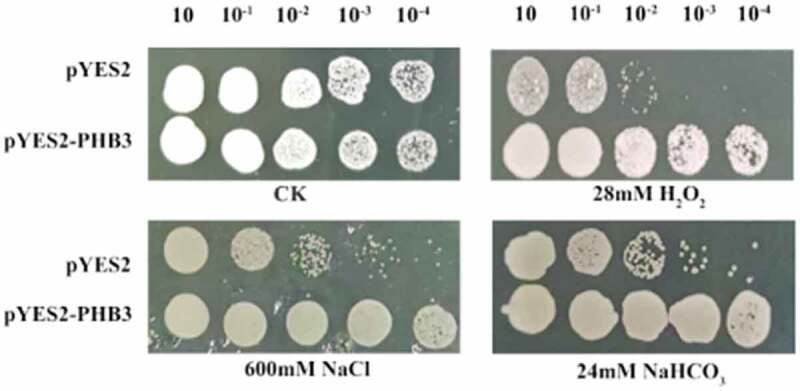
Growth of *slPHB3 t*ransgenic yeast cells under salt stress. Ten-fold dilutions of yeast cells containing pYES2 (upper line) and pYES2-*slPHB3* vector (lower line) were spotted onto solid YPG media supplemented with the indicated stresses. No treatment is a control (CK).

The yeast cells growth under various abiotic stresses were studied (Figure 4). The *slPHB3*-transgenic lines grew normally on the medium, while the control plants did not growth in the presence of 1 mM NaCl, 24 mM NaHCO_3_ or 3.2 mM H_2_O_2_. The *slPHB3*-transgenic lines grew better than the control.

### Stress tolerance of the transgenic tobacco

**Figure 5.**
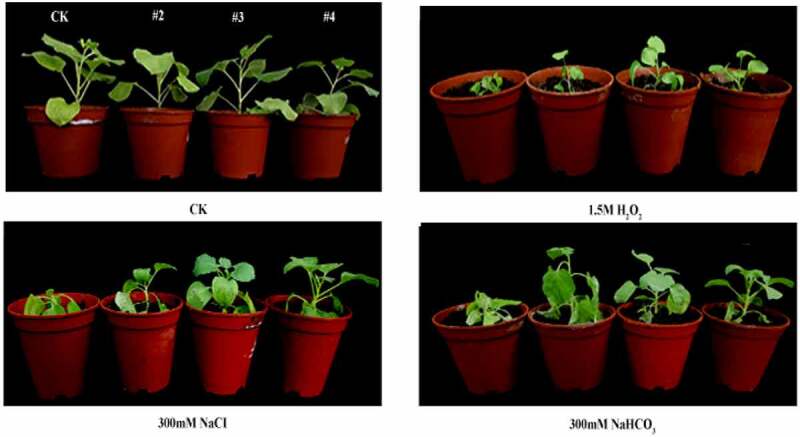
The relative stress tolerance of wild-type and transgenic plants (2, # 3, # 4) in reproduction stage was studied. The plants grown on the soil with 1.5 M H_2_O_2_, 300 mM NaCl or 300 mM NaHCO_3_ or without (CK).

The plants grew well in the medium without stress. The transgenic plants severely lost water and wilted than wild type after stress treatment (Figure 5). There was no significant difference in fresh weight under CK conditions. To the opposite, under stress treatments, the fresh weight of over-expressed plants was larger than those of wild type, which generally grew better and suffered less damage (Table 1).

**Table 1.** Fresh weigh (mg) of tobacco under different stress treatments. Results are present as means of 10 plants. CK, control; WT, wild type; #2, #3 and #4 are *slPHB3* transgenic plants

Fresh weigh (mg)	WT	#2	#3	#4
CK	31.6	31.68	31.88	31.61
1.5 M H_2_O_2_	20.94	25.14	24.55	26.41
300 mM NaCl	20.91	24.64	26.75	27.81
300 mM NaHCO_3_	20.10	26.01	25.95	23.84

### *Expression of salt stress-related genes(*NHX1 *and* SOS1*) in tobacco*

**Figure 6.**
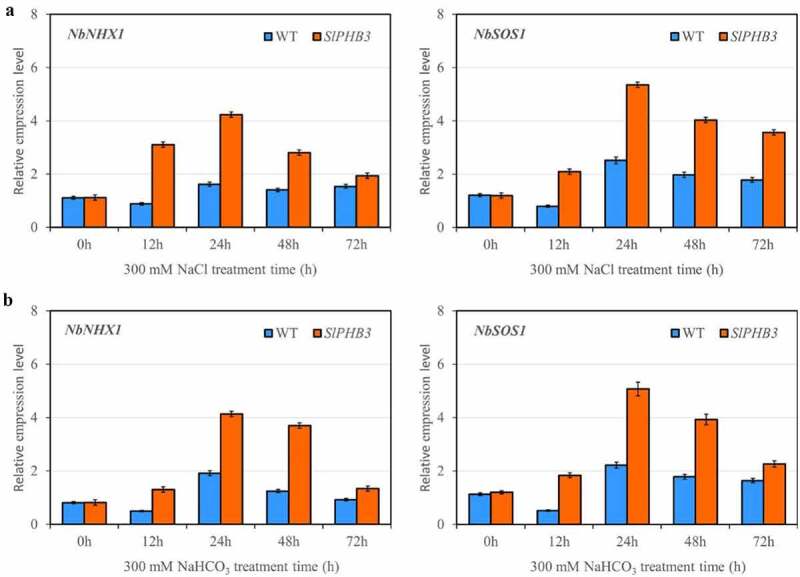
Quantitative qRT-PCR determination of representative gene expression in leaves. Relative expression level of *slPHB3* in leaves of wildtype tobacco and transgenic tobacco seedlings exposed to 300 mM NaCl (a) or 300 mM NaHCO_3_ (b), values are mean ± SD, n = 3. Tublin was used as an internal control.

In order to further investigate the mechanisms of *slPHB3* overexpression plants enhanced salt tolerance, expression levels of salt-related marker genes (*NHX1* and *SOS1*) in salt response pathways were determined. Under salt condition, *NHX1* and *SOS1* showed steadily higher expression in overexpression lines than in WT (Figure 6).

### Effects of treatments on H_2_O_2_ content in the transgenic tobacco

**Figure 7.**
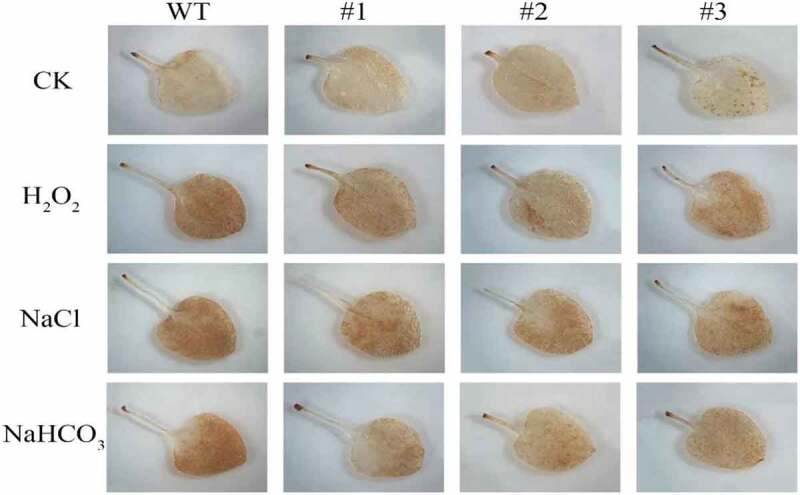
H_2_O_2_ accumulation in leaves was detected by DAB staining. DAB staining in leaves in wild type and transgenic plants (2, # 3, # 4) under 1.5 M H_2_O_2_, 300 mM NaCl or 300 mM NaHCO_3_ stresses.

Hydrogen peroxide in tobacco leaves was detected in situ using 3, 3′-Diaminobenzidine (DAB) histochemical staining method. The color of the leaf showed no difference between WT and *slPHB3*-transgenic plants without stresses. The color of the WT leaf was darker than that of the transgenic line leaves under different stress (Figure 7). H_2_O_2_ accumulation in wildtype or transgenic plants was detected under stress conditions. The H_2_O_2_ content in the transgenic line was lower than that of the WT plant after 48 h treatment (Table 2) which indicated that *slPHB3* increased the H_2_O_2_ scavenging function of the transgenic plants.

**Table 2.** H_2_O_2_ content in leaves of wild type and transgenic Arabidopsis under different stresses was measured with Plant H_2_O_2_ Kit. Data are means of three replicates. WT, wild type; #2, #3 and #4 are *slPHB3* transgenic plants

ROS (content)	WT	#2	#3	#4
CK	2.56	2.68	2.88	2.61
1.5 M H_2_O_2_	4.34	3.14	3.55	3.41
300 mM NaCl	4.91	3.64	3.75	3.81
300 mM NaHCO_3_	5.41	4.01	3.95	3.84

### *Screening of* slPHB3 *interacting protein by yeast two-hybrid*

The total of 17 positive gene were obtained from colonies grown on SD/-Trp-Leu-His-Ade+X-α-gal+AbA solid medium turned blue. BLAST these colonies in NCBI, 17 genes were successfully compared (Table 1), ATP-dependent protease La regulates protein stability, Lon hydrolyzed protein impaired seedling production. The glycosyl hydrolase is related to low temperature stress and osmotic stress, P-loop containing nucleoside triphosphate hydrolases, HSP and PAO4 are related to temperature stress. ATP synthase subunit beta is related to cold stress and oxidative stress. ATP synthase epsilon chain and TIF7 are related to salt stress, NAC13 is related to salt and drought stress. Glycine decarboxyla plays a major role in photorespiration. Non-intrinsic ABC protein family functions are related to accumulation of metal ions and stability of chloroplast structure. ATNAP7 is an essential ATP binding site for embryo development. PAO5 is highly responsive to drought stress. Salt induced the expression of *SOT12* gene. Tom3 have a specific role in the transport of plant iron carriers. Proline-rich nuclear receptor modulates transcriptional activation of multiple nuclear receptors.

## Materials and methods

### Plant materials and growth conditions

*Salix linearistipularis* and tobacco plants were grown in a controlled growth chamber at 24 ± 2°C with a 16 h light/8 h dark cycle.

### *Cloning and bioinformatics analysis of* slPHB3 *gene*

Total RNA from leaves of *Salix linearistipularis* was isolated using RNeasy Plant Mini Kit (Qiagen, Hilden, Germany), first-strand cDNA was synthesized by reverse transcribing 500 ng of total RNA with using Prime-Script Reverse Transcriptase (Takara, Tokyo, Japan). The forward *slPHB3F* and reverse primer *slPHB3*R (slPHB3F: ATGGGTAGCAGCCAAGCAGC, slPHB3R: TCAACGGTTTGCATTCAGGG) were designed according to the *Salix linearistipularis* transcriptome data. The PCR products were ligated to pMD18-T vector (Takara, Tokyo, Japan) and sequenced. The homologous amino acid sequence of slPHB3 protein were compared using DNAMAN software, and the phylogenetic tree was constructed using MEGA7 software.

### *Real-time quantitative PCR (RT-qPCR) analysis for* slPHB3 *expression*

*Salix linearistipularis* seeds were sown onto 1/2 MS medium. The seedlings of one-month age were exposed to 3 mM H_2_O_2_, 150 mM NaCl and 5 mM NaHCO_3_ treatments for 0, 6, 12, 24, 36 or 72 h, respectively. The *slPHB3* expression in leaves under treatments was examined by RT-qPCR analysis. Total RNA was isolated from leaves of *Salix linearistipularis* and cDNA was synthesized. Subsequently RT-qPCR analyses were carried out by SYBR green (Takara, Tokyo, Japan) and IQ5 real-time PCR equipment (Bio-Rad, Hercules, CA, USA) with the steps: 95°C for 30s, 30 cycles of 95°C for 5s, and 55°C for 30s. The next steps were added to meltcurve analysis: 95°C for 15s, followed by continuously increased from 60°C to 95°C. The *SaActin* gene expression was used as control. The forward primer sequence was SaActin-F: GGTAACATTGTGCTCAGTGGTGG and reverse primer sequence was SaActin-R: AACGACCTTAATCTTCATGCTGC. All tests were repeated in triplicate.

### Construction of yeast expression vectors and transformation

The coding region of the *slPHB3* gene was PCR amplified with *Bam*HI sense primer 5’- GGATCCATGGGTAGCAGCCAAGCAGC-3’ and *Xho*I antisense primer 5’-CTCGAG TCAACGGTTTGCATTCAGGG −3’. The PCR amplified fragments were digested with *Bam*HI and *Xho*I, then subcloned to the same site of the pYES2 expression vector (Clontech, Tokyo, Japan) resulting in pYES2-*slPHB3*. Then transformed into the competent yeast strain INVSC1 for protein expression in Yeast. Transgenic yeast cells (OD_600_ = 0.5) containing pYES2-*slPHB3* and pYES_2_ (control) with serial dilutions (10, 10^−1^, 10^−2^, 10^−3^ and 10^−4^) were spotted onto YPD agar plates supplemented with 3.2 mM H_2_O_2_, 1 M NaCl or 24 mM NaHCO_3_, respectively.

### *Acquisition of* slPHB3 *overexpressed tobacco transgenic lines*

The *slPHB3* PCR amplified fragment with added with *Bam*H I and *Xho* I restriction enzymes was obtained by *slPHB3Bam*H I-F and reverse primer *slPHB3Xho* I-R and ligase into pBI121 vector plasmid, the plasmid DNAs of pBI121-*slPHB3* was transformed into the *Agrobacterium tumefaciens* strain *EHA105* (Takara, Tokyo, Japan), and the tobacco was infected with agrobacterium. The independent transgenic lines were obtained and verified the expression of *slPHB3* by RT-qPCR, all temples were tested in triplicate, then used for further analyses.

Wild type and transgenic seed were planted to pots containing nutrient-rich soil. After growing up for two months, the pots were irrigated with 50 mL solution of 1.5 M H_2_O_2_, 300 mM NaCl or 300 mM NaHCO_3_ every 4 days. The pots were covered with a breathable plastic cover to minimize evaporation and keep the concentration of the solution from changing too much. Images of the plants were taken after 12 days of treatment.

### *RT-qPCR analysis for* NHX1 *and* SOS1 *expression in tobacco*

The expression levels of the *NHX1* (Accession number: JX987081.1), and *SOS1*(Accession number: JK739016.1) were analyzed using qRT-PCR, Total RNA was isolated from leaf samples of 300 mM NaCl or 300 mM NaHCO_3_ treated tobacoo plants. The relative levels of the transcript accumulated for the *NHX1* (NbNHX1qPCR -F: 5′ - AGGCACGCTTTTGCAACTTT -3ʹ,NbNHX1qPCR-R:5ʹ-ACACTGCGCCTCTCATCAAA-3ʹ), and *SOS1* genes (NbSOS1qPCR-F:5ʹ-Acagctagtttttctcatcaa-3ʹ, NbSOS1qPCR-R: 5ʹ-tgaccagttcgttccacatc-3ʹ) were normalized to actin (NbActin-F: 5ʹ-tggtcgtaccaccggtattgtgtt-3ʹ, NbActin-R:5ʹ- tcacttgcccatcaggaagctcat-3ʹ).^20^

### Reaction to H_2_O_2_ stress in transgenic tobacco plants

One-month-old WT and transgenic plants were treated without (control) or with each of 1.5 M H_2_O_2_, 300 mM NaCl or 300 mM NaHCO_3_ respectively for 48 h. H_2_O_2_ accumulation in plant leaves was visualized by histochemical staining with 3, 3′-Diaminobenzidine (DAB). The treated leaves were immersed in 1 mg·mL^−1^ DAB solution (Solarbio, Beijing, China), incubated in the dark at room temperature, after DAB solution incubated, the samples chlorophyll was removed using absolute ethanol, the leaf was photographed with a microscopy (Olympus). The H_2_O_2_ content was also measured using Plant H_2_O_2_ ELISA Kit (America Rapid Bio).

### *Screening of* slPHB3 *interacting protein by yeast two-hybrid*

*slPHB3* was cloned into pGADT7 vector, the recombinant plasmids pGADT7-*slPHB3* were identified by double enzyme digestion, then they were transformed into Y_2_HGold strain, the proteins interacting with pGADT7-*slPHB3* were screened from *Salix linearistipularis* cDNA library. Yeast DNA was extracted and sequenced to obtain the interaction gene and protein sequences.

## Discussion

A *slPHB3* gene was cloned from *Salix linearistipularis* to develop the *Salix linearistipularis* resistance mechanism. The *slPHB3* expression level increased under 3 mM H_2_O_2_, 125 mM NaCl and 5 mM NaHCO_3_ stress (Figure 3). This means the stresses influenced the *slPHB3* gene expression level in *Salix linearistipularis* leaves.

In the yeast resistance analysis, the growth of the transgenic strain was better than that of the control under 1 mM NaCl, 24 mM NaHCO_3_ or 3.2 mM H_2_O_2_ medium treatment, indicating that *slPHB3* gene expression increased yeast resistant to stress. Yeast belongs to Eukaryote, which shows that *slPHB3* expression in yeast can improve the saline resistance of eukaryote.

Under the 2 M H_2_O_2_, 300 mM NaCl or 300 mM NaHCO_3_ stress, the fresh weight of over-expressed plants was larger than those of wild type, the wild-type plants died, while the transgenic plants survived (Figure 2d). The results showed that overexpression *slPHB3* can improved tobacco stresses resistance.

Molecular mechanisms underlying *slPHB3* functioning under salt conditions have not been completely elucidated, *NHX1* and *SOS1* expression to regulate Na^+^ ions toxicity that leads to enhanced salinity stress tolerance in tomato plants,^21^ BvSOS1 and BvNHX1 played vital roles in tetraploid sugar beet cultivar which exhibited more tolerant to salinity than diploid sugar beet,^22^ two pomegranate cultivars study finding that difference between cultivars in salt tolerance is associated with transcriptional regulation of *SOS1* and *NHX1* genes.^23^ In this study, we analyzed the expression of two salt-related marker gene (*NHX1* and *SOS1*) in tobacco, both *NHX1* and *SOS1* expression higher in overexpression lines than that in WT (Figure 6), NHX1 and SOS1 have a high capacity for scavenging ROS under salt stress.^17–19^ The increased transcript level of *NHX1* and *SOS1* in overexpression plants under salinity stress may increase plant tolerance to salt by improving ROS scavenging capacity.

In important number of ROS is produced under the stresses.^24^ Upon *PHB3* loss-of-function, the ROS contents will be out of homeostasis.^11^ Compared to WT, H_2_O_2_ was accumulated more in the *PHB3* mutant root meristem.^14^ These study showed that PHB3 was relation to ROS contents in plant. In this study, the content of ROS in overexpressing *slPHB3* tobacco was significantly higher than that in wild-type tobacco. The results showed that tobacco overexpressing *slPHB3* could reduce the content of ROS and eliminate the effect of excessive ROS on plants to improved tobacco stresses resistance.

PHB3 also interacts with a variety of other proteins. In mitochondria, PHB3 forms complexes with other PHB proteins. The formation of the atPHB3-ICS1 complex stabilizes ICS1 to promote SA production.^9^ In this research, 17 sequences were successfully matched (Table 3). The function of P-loop containing nucleoside triphosphate hydrolases is related to temperature changes.^25^ The function of glycosyl hydrolase family is related to glycosylation.^26^ The glycosyl hydrolase 18 (GH18) and GH19 families are also related to low temperature stress and osmotic stress,^27^ ATP-dependent protease La is involved in the degradation of abnormally folded proteins and specific regulatory proteins, and regulates protein stability.^28^ Lack of Lon hydrolyzed protein causes plant growth retardation and impaired seedling production.^29^ ATP Synthase subunit Beta family is involved in REDOX reactions, mediates protein interactions, and is associated with cold stress.^30,31^ Proteome analysis of Date Palm showed that the α and β subunits of ATP synthase changed significantly under salt stress and drought stress.^32^ Alpha/beta-hydrolases superfamily functions to catalyze the hydrolysis of ester bonds between fatty acids and glycerol.^33^ It was involved in delaying the senescence of strawberry fruit at low temperature.^34^ HSP family functions are related to temperature changes.^35^ However, under NaCl stress, the root-cap length of OSHSP40 transgenic seedlings was significantly shorter than that of wild-type seedlings. The results showed that HSP40 was related to salt stress.^36^ The TIFY family may play crucial and divergent roles in phytohormone crosstalk and plant defense.^37^ The TIFY gene responds to abiotic stresses such as jasmonic acid (JA) and salt and drought.^38^ Glycine decarboxyla plays a major role in photorespiration. Glycine decarboxylase and other proteins increase CO_2_ assimilation, vegetative biomass, and seed yield in Arabidopsis thaliana. AtGLDP1 is involved in the transition of C-3 through C-2 to C4 photosynthesis.^39^ Non-intrinsic ABC protein family functions are related to accumulation of metal ions and stability of chloroplast structure.^40^ ATNAP7 is an essential ATP binding site for Arabidopsis embryo development.^41^ PAO family functions play a major role in PA catabolism. ScPAO5 is highly responsive to drought stress, while ScPAO1 and CspAO2 are sensitive to changes in nitrogen nutrition.^42^ Transcription level of PaO4 in tomato leaves increased in response to heat stress and cold stress.^43^ Glycoprotein effect on rice seedlings salt tolerance.^44^ loss of NatB function increased plant sensitivity toward osmotic and high-salt stress, indicating that NatB is required for tolerance of these abiotic stress.^45^ Salt, osmotic stress and hormone treatment strongly induced the expression of Atsot12 gene.^46^ Tom3 is thought to have a specific role in the transport of plant iron carriers.^47^ Proline-rich nuclear receptor coactivator participates regulatory protein that modulates transcriptional activation of multiple nuclear receptors.^48^

**Table 3.** Information on the putative slPHB3-interacting proteins identified by the Y_2_H system

	Gene family
1	P-loop containing nucleoside triphosphate
2	hydrolases glycosyl hydrolase
3	ATP-dependent protease La
4	ATP synthase subunit beta
5	ATP synthase epsilon chain
6	alpha/beta-Hydrolases superfamily
7	DNAJ heat shock family protein
8	TIFY domain/Divergent
9	glycine decarboxyla
10	non-intrinsic ABC protein
11	polyamine oxidase
12	ARABIDOPSIS P-GLYCOPROTEIN
13	Acyl-CoA N-acyltransferases (NAT) superfamily protein
14	NAC domain protein
15	SOT
16	TOM
17	Proline-rich nuclear receptor coactivator

Some NAC genes have been identified as candidates for breeding programmer to improve drought resistance in crops.^49^ In maize, 13 SNAC transcripts in the SNAC subfamily were responsive to drought stress, and almost all of them in roots and 11 in leaves were upregulated under drought stress.^50^ The NAC-type transcription factor CaNAC46 from Capsicum annuum regulates the salt and drought tolerance of transgenic Arabidopsis thaliana.^51^

**Figure 8.**
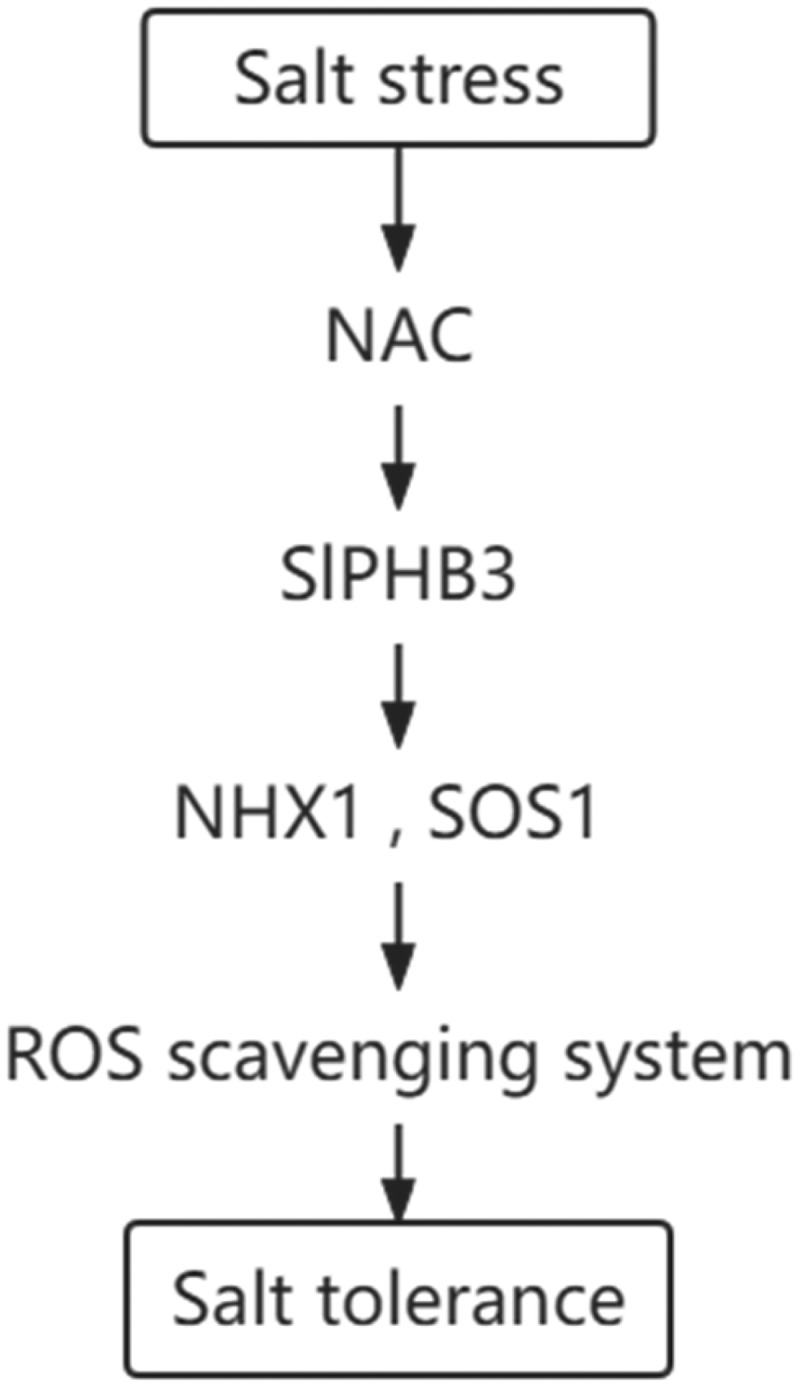
Model of the regulatory mechanisms of the *slPHB3* gene in response to the tolerance to salt stresses. Salt stress increased expression of NAC, NAC might induce the increase of *slPHB3* expression, *slPHB3* induceed the increase of *NHX1* and *SOS1* expression, *NHX1* and *SOS1* genes enhanced the scavenging effect on ROS, which resulted in improved tolerance to salt stresses.

In our study, H_2_O_2_ content were significantly lower in *slPHB3*-overexpressing tobacco lines comparing to those in WT plants under salt stresses, overexpression of *slPHB3* likely improved salt tolerance through the ROS scavenging system pathways. We identified NAC proteins interacting with slPHB3 by yeast two-hybrid technique. Consistent with these findings, we hypothesized a molecular pathway that the increase of NAC gene expression under salt treatment. NAC might induce the increase of *slPHB3* expression, *slPHB3* may increase the expression of *NHX1* and *SOS1*, NHX1 and SOS1 improved ROS scavenging capacity. In this pathway, slPHB3 enhanced the scavenging effect on ROS and improved plant tolerance (Figure 8).

## Conclusion

The growth of recombinant yeast cells was better than that of the control group under the stress treatment. Comparison between *slPHB3* transgenic tobacco and wild-type tobacco showed that the transgenic plants had higher salt tolerance and oxidation resistance than the wild-type plants. Expression of *NHX1* and *SOS1* in transgenic tobacoo are higher in that in wild-types. Through the screening of slPHB3 interaction proteins, 10 of the 17 genes were related to abiotic stress, indicating that *PHB3* gene plays a role in plant stress resistance.

## References

[1] Atkinson NJ, Urwin PE. The interaction of plant biotic and abiotic stresses: from genes to the field. J Exp Bot. 2012;63(10):3523–10. doi:10.1093/jxb/ers100. 22467407

[2] Ishida TA, Nara K, Ma SR, Takano T, Liu SK. Ectomycorrhizal fungal community in alkaline-saline soil in northeastern China. Mycorrhiza. 2009;19(5):329–335. doi:10.1007/s00572-008-0219-9. 19104846

[3] Nan GX, Zhang Y, Li S, Lee I, Takano T, Liu SK. NaCl stress-induced transcriptomics analysis of Salix linearistipularis (syn. Salix mongolica). J Biol Res. 2016;23:14. doi:10.1186/s40709-016-0038-7. PMC4772304 26933650

[4] McClung JK, Danner DB, Stewart DA, Smith JR, Schneider EL, Lumpkin CK, Dell’Orco RT, Nuell MJ. Isolation of a cDNA that hybrid selects antiproliferative mRNA from rat liver. Biochem Biophys Res Commun. 1989;164(3):1316–1322. doi:10.1016/0006-291x(89)91813-5. 2480116

[5] Di C, Xu WY, Su Z, Yuan JS. Comparative genome analysis of PHB gene family reveals deep evolutionary origins and diverse gene function. BMC Bioinform. 2010;11(S6):15. doi:10.1186/1471-2105-11-s6-s22. PMC3026370 20946606

[6] Thuaud F, Ribeiro N, Nebigil CG, Desaubry L. Prohibitin ligands in cell death and survival: mode of action and therapeutic potential. Chem Biol. 2013;20(3):316–331. doi:10.1016/j.chembiol.2013.02.006. 23521790 PMC7111013

[7] Merkwirth C, Dargazanli S, Tatsuta T, Geimer S, Lower B, Wunderlich FT, von Kleist-retzow JC, Waisman A, Westermann B, Langer T. Prohibitins control cell proliferation and apoptosis by regulating OPA1-dependent cristae morphogenesis in mitochondria. Genes Dev. 2008;22(4):476–488. doi:10.1101/gad.460708. 18281461 PMC2238669

[8] Merkwirth C, Langer T. Prohibitin function within mitochondria: essential roles for cell proliferation and cristae morphogenesis. Biochim Biophys Acta-Mol Cell Res. 2009;1793(1):27–32. doi:10.1016/j.bbamcr.2008.05.013. 18558096

[9] Seguel A, Jelenska J, Herrera-Vasquez A, Marr SK, Joyce MB, Gagesch KR, Shakoor N, Jiang SC, Fonseca A, Wildermuth MC, et al. PROHIBITIN3 forms complexes with ISOCHORISMATE SYNTHASE1 to regulate stress-induced salicylic acid biosynthesis in arabidopsis. Plant Physiol. 2018;176(3):2515–2531. doi:10.1104/pp.17.00941. 29438088 PMC5841719

[10] Wang Y, Ries A, Wu KT, Yang A, Crawford NM. The arabidopsis prohibitin gene phb3 functions in nitric oxide-mediated responses and in hydrogen peroxide-induced nitric oxide accumulation. Plant Cell. 2010;22(1):249–259. doi:10.1105/tpc.109.072066. 20068191 PMC2828708

[11] Huang RH, Yang CW, Zhang SC. The Arabidopsis PHB3 is a pleiotropic regulator for plant development. Plant Signal Behav. 2019;14(11):5. doi:10.1080/15592324.2019.1656036. PMC6804698 31429630

[12] Van Aken O, Pecenkova T, van de Cotte B, De Rycke R, Eeckhout D, Fromm H, De Jaeger G, Witters E, Beemster GTS, Inze D, et al. Mitochondrial type-I prohibitins of Arabidopsis thaliana are required for supporting proficient meristem development. Plant J. 2007;52(5):850–864. doi:10.1111/j.1365-313X.2007.03276.x. 17883375

[13] Woo SP, Ramasamy K, Joe MM, Sa T. EPS production, PHB accumulation and abiotic stress endurance of plant growth promoting methylobacterium strains grown in a high carbon concentration. Korean Journal of Soil Science & Fertilizer. 2012;45(4):572–581. doi:10.7745/kjssf.2012.45.4.572.

[14] Kong XP, Tian HY, Yu QQ, Zhang F, Wang R, Gao S, Xu WH, Liu JJ, Shani E, Fu CX, et al. PHB3 Maintains Root Stem Cell Niche Identity through ROS-Responsive AP2/ERF Transcription Factors in Arabidopsis. Cell Rep. 2018;22(5):1350–1363. doi:10.1016/j.celrep.2017.12.105. 29386120

[15] Zhang WD, Wang P, Bao ZLT, Ma Q, Duan LJ, Bao AK, Zhang JL, Wang SM. SOS1, HKT1;5, and NHX1 synergistically modulate Na+ homeostasis in the halophytic grass puccinellia tenuiflora. Front Plant Sci. 2017a;8:8. doi:10.3389/fpls.2017.00576. 28450879 PMC5390037

[16] Chen CZ, Lv XF, Li JY, Yi HY, Gong JM. Arabidopsis NRT1.5 is another essential component in the regulation of nitrate reallocation and stress tolerance. Plant Physiol. 2012;159(4):1582–1590. doi:10.1104/pp.112.199257. 22685171 PMC3425198

[17] Zhou Y, Yin XC, Wan SM, Hu YP, Xie Q, Li RM, Zhu BB, Fu SP, Guo JC, Jiang XY. The sesuvium portulacastrum plasma membrane Na+/H+ Antiporter SpSOS1 complemented the salt sensitivity of transgenic arabidopsis sos1 mutant plants. Plant Mol Biol Rep. 2018;36(4):553–563. doi:10.1007/s11105-018-1099-6.

[18] Feki K, Tounsi S, Masmoudi K, Brini F. The durum wheat plasma membrane Na+/H+ antiporter SOS1 is involved in oxidative stress response. Protoplasma. 2017;254(4):1725–1734. doi:10.1007/s00709-016-1066-8. 28013410

[19] Zhang YD, Deng GF, Fan WJ, Yuan L, Wang HX, Zhang P. NHX1 and eIF4A1-stacked transgenic sweetpotato shows enhanced tolerance to drought stress. Plant Cell Rep. 2019;38(11):1427–1438. doi:10.1007/s00299-019-02454-6. 31396684

[20] Ha JH, Jang HA, Moon KB, Baek KH, Choi GJ, Choi D, Cho HS, Kwon SY, Jeon JH, Oh SK, et al. Nicotiana benthamiana matrix metalloprotease 1 (NMMP1) gene confers disease resistance to Phytophthora infestans in tobacco and potato plants. J Plant Physiol. 2017;218:189–195. doi:10.1016/j.jplph.2017.08.010. 28888160

[21] Rao YR, Ansari MW, Sahoo RK, Wattal RK, Tuteja N, Kumar VR. Salicylic acid modulates ACS, NHX1, sos1 and HKT1;2 expression to regulate ethylene overproduction and Na+ ions toxicity that leads to improved physiological status and enhanced salinity stress tolerance in tomato plants cv. Pusa Ruby Plant Signal Behav. 2021;16(11):9. doi:10.1080/15592324.2021.1950888. PMC8526040 34252347

[22] Li SJ, Wu GQ, Lin LY. AKT1, HAK5, SKOR, HKT1;5, SOS1 and NHX1 synergistically control Na+ and K+ homeostasis in sugar beet (Beta vulgaris L.) seedlings under saline conditions. J Plant Biochem Biotechnol. 2021;14. doi:10.1007/s13562-021-00656-2.

[23] Calzone A, Cotrozzi L, Pellegrini E, Lorenzini G, Nali C, Maathuis F. Can the transcriptional regulation of NHX1, SOS1 and HKT1 genes handle the response of two pomegranate cultivars to moderate salt stress? Salt-tolerance of two pomegranate cultivars. Sci Hortic. 2021;288:8. doi:10.1016/j.scienta.2021.110309.

[24] Jubany-Mari T, Munne-Bosch S, Alegre L. Redox regulation of water stress responses in field-grown plants. Role of hydrogen peroxide and ascorbate. Plant Physiol Biochem. 2010;48(5):351–358. doi:10.1016/j.plaphy.2010.01.021. 20199867

[25] Zhao L, Vecchi G, Vendruscolo M, Korner R, Hayer-Hartl M, Hartl FU. The Hsp70 chaperone system stabilizes a thermo-sensitive subproteome in E. coli. Cell Rep. 2019;28(5):1335-+. doi:10.1016/j.celrep.2019.06.081. 31365874

[26] Kotik M, Brodsky K, Halada P, Javurkova H, Pelantova H, Konvalinkova D, Bojarova P, Kren V. Access to both anomers of rutinosyl azide using wild-type rutinosidase and its catalytic nucleophile mutant. Catal Commun. 2021;149:5. doi:10.1016/j.catcom.2020.106193.

[27] Chen JJ, Piao YL, Liu YM, Li XN, Piao ZY. Genome-wide identification and expression analysis of chitinase gene family in Brassica rapa reveals its role in clubroot resistance. Plant Sci. 2018;270:257–267. doi:10.1016/j.plantsci.2018.02.017. 29576079

[28] Tsitsekian D, Daras G, Alatzas A, Templalexis D, Hatzopoulos P, Rigas S. Comprehensive analysis of Lon proteases in plants highlights independent gene duplication events. J Exp Bot. 2019;70(7):2185–2197. doi:10.1093/jxb/ery440. 30590727 PMC6460959

[29] Rigas S, Daras G, Tsitsekian D, Hatzopoulos P. The multifaceted role of Lon proteolysis in seedling establishment and maintenance of plant organelle function: living from protein destruction. Physiol Plant. 2012;145(1):215–223. doi:10.1111/j.1399-3054.2011.01537.x. 22023720

[30] Yang JH, Williams D, Kandiah E, Fromme P, Chiu PL. Structural basis of redox modulation on chloroplast ATP synthase. Commun Biol. 2020;3(1):12. doi:10.1038/s42003-020-01221-8. 32879423 PMC7468127

[31] Zhang ZY, Li JJ, Pan YH, Li JL, Zhou L, Shi HL, Zeng YW, Guo HF, Yang SM, Zheng WW, et al. Natural variation in CTB4a enhances rice adaptation to cold habitats. Nat Commun. 2017b;8:13. doi:10.1038/ncomms14788. 28332574 PMC5376651

[32] El Rabey HA, Al-Malki AL, Abulnaja KO, Rohde W. Proteome analysis for understanding abiotic stress (salinity and drought) tolerance in date palm (Phoenix dactylifera L.). Int J Genomics. 2015;2015:11. doi:10.1155/2015/407165. PMC4488584 26167472

[33] Zan XY, Cui FJ, Sun JN, Zhou S, Song YD. Novel dual-functional enzyme lip10 catalyzes lipase and acyltransferase activities in the oleaginous fungus *Mucor circinelloides*. J Agric Food Chem. 2019;67(47):13176–13184. doi:10.1021/acs.jafc.9b05617. 31690075

[34] Xu XB, Ma XY, Lei HH, Yin LL, Shi XQ, Song HM. MicroRNAs play an important role in the regulation of strawberry fruit senescence in low temperature. Postharvest Biol Technol. 2015;108:39–47. doi:10.1016/j.postharvbio.2015.05.006.

[35] Li GL, Zhao H, Guo HB, Wang Y, Cui XP, Li H, Xu BH, Guo XQ. Analyses of the function of DnaJ family proteins reveal an underlying regulatory mechanism of heat tolerance in honeybee. Sci Total Environ. 2020a;716:15. doi:10.1016/j.scitotenv.2020.137036. 32059293

[36] Wang X, Zhang H, Shao LY, Yan X, Peng H, Ouyang JX, Li SB. Expression and function analysis of a rice OsHSP40 gene under salt stress. Genes Genom. 2019;41(2):175–182. doi:10.1007/s13258-018-0749-2. 30298358

[37] Liu X, Zhao CB, Yang LM, Zhang YY, Wang Y, Fang ZY, Lv HH. Genome-wide identification, expression profile of the TIFY gene family in brassica oleracea var. capitata, and their divergent response to various pathogen infections and phytohormone treatments. Genes. 2020;11(2):19. doi:10.3390/genes11020127. PMC7073855 31991606

[38] Yang YX, Ahammed GJ, Wan CP, Liu HJ, Chen RR, Zhou Y. Comprehensive analysis of TIFY transcription factors and their expression profiles under jasmonic acid and abiotic stresses in watermelon. Int J Genomics. 2019;2019:13. doi:10.1155/2019/6813086. PMC6791283 31662958

[39] Adwy W, Laxa M, Peterhansel C. A simple mechanism for the establishment of C-2-specific gene expression in Brassicaceae. Plant J. 2015;84(6):1231–1238. doi:10.1111/tpj.13084. 26603271

[40] Shimoni-Shor E, Hassidim M, Yuval-Naeh N, Keren N. Disruption of Nap14, a plastid-localized non-intrinsic ABC protein in Arabidopsis thaliana results in the over-accumulation of transition metals and in aberrant chloroplast structures. Plant Cell Environ. 2010;33(6):1029–1038. doi:10.1111/j.1365-3040.2010.02124.x. 20132520

[41] Xu XM, Moller SG. AtNAP7 is a plastidic SufC-like ATP-binding cassette/ATPase essential for Arabidopsis embryogenesis. Proc Natl Acad Sci U S A. 2004;101(24):9143–9148. doi:10.1073/pnas.0400799101. 15184673 PMC428487

[42] Li MS, Lu J, Tao MM, Li MR, Yang H, Xia EH, Chen Q, Wan XC. Genome-Wide Identification of Seven Polyamine Oxidase Genes inCamellia sinensis(L.) and Their Expression Patterns Under Various Abiotic Stresses. Front Plant Sci. 2020b;11:12. doi:10.3389/fpls.2020.544933. 33013966 PMC7500180

[43] Upadhyay RK, Fatima T, Handa AK, Mattoo AK. Polyamines and their biosynthesis/catabolism genes are differentially modulated in response to heat versus cold stress in tomato leaves (Solanum lycopersicumL.). Cells. 2020;9(8):20. doi:10.3390/cells9081749. PMC7465501 32707844

[44] Fang LJ, Fu XO, Ye QS, Wu T, Wang ZC, Chen X, Zhu ZK, Zhang XF, Fu CK. Effects of P-glycoprotein inhibitor and elicitor on the salt tolerance of rice seedlings. J Integr Plant Biol. 2007;49(5):588–597. doi:10.1111/j.1744-7909.2007.00440.x.

[45] Huber M, Bienvenut WV, Linster E, Stephan I, Armbruster L, Sticht C, Layer D, Lapouge K, Meinnel T, Sinning I, et al. NatB-Mediated N-terminal acetylation affects growth and biotic stress responses. Plant Physiol. 2020;182(2):792–806. doi:10.1104/pp.19.00792. 31744933 PMC6997699

[46] Baek D, Pathange P, Chung JS, Jiang JF, Gao LQ, Oikawa A, Hirai MY, Saito K, Pare PW, Shi HZ. A stress-inducible sulphotransferase sulphonates salicylic acid and confers pathogen resistance in Arabidopsis. Plant Cell Environ. 2010;33(8):1383–1392. doi:10.1111/j.1365-3040.2010.02156.x. 20374532

[47] Nozoye T, Nagasaka S, Kobayashi T, Sato Y, Uozumi N, Nakanishi H, Nishizawa NK. The Phytosiderophore Efflux Transporter TOM2 Is Involved in Metal Transport in Rice. J Biol Chem. 2015;290(46):27688–27699. doi:10.1074/jbc.M114.635193. 26432636 PMC4646018

[48] Zhou D, Quach KM, Yang C, Lee SY, Pohajdak B, Chen S. PNRC: a proline-rich nuclear receptor coregulatory protein that modulates transcriptional activation of multiple nuclear receptors including orphan receptors SF1 (steroidogenic factor 1) and ERRalpha1 (estrogen related receptor alpha-1). Molecular Endocrinology (Baltimore, Md). 2000;14(7):986–998. doi:10.1210/me.14.7.986. 10894149

[49] Sanjari S, Shirzadian-Khorramabad R, Shobbar ZS, Shahbazi M. Systematic analysis of NAC transcription factors’ gene family and identification of post-flowering drought stress responsive members in sorghum. Plant Cell Rep. 2019;38(3):361–376. doi:10.1007/s00299-019-02371-8. 30627770

[50] Li L, Ma YW, Zhang SH, Hao ZF, Li XH. Zea mays NAC transcription factor family members: their genomic characteristics and relationship with drought stress. Res J Biotechnol. 2015;10:63–77.

[51] Ma J, Wang LY, Dai JX, Wang Y, Lin D. The NAC-type transcription factor CaNAC46 regulates the salt and drought tolerance of transgenic Arabidopsis thaliana. BMC Plant Biol. 2021;21(1):11. doi:10.1186/s12870-020-02764-y. 33407148 PMC7788707

